# Hallmarks of cellular senescence: biology, mechanisms, regulations

**DOI:** 10.1038/s12276-025-01480-7

**Published:** 2025-07-10

**Authors:** Amir Ajoolabady, Domenico Pratico, Suhad Bahijri, Jaakko Tuomilehto, Vladimir N. Uversky, Jun Ren

**Affiliations:** 1 National Clinical Research Center for Interventional Medicine, Shanghai, 200032 China; 2 Alzheimer’s Center at Temple, Lewis Katz School of Medicine, Temple University, Philadelphia, PA USA; 3 Diabetes Research Group, King Abdulaziz University, Jeddah, Saudi Arabia; 4 Department of Public Health, University of Helsinki, Helsinki, Finland; 5 Health Promotion Unit, Finnish Institute for Health and Welfare, Helsinki, Finland; 6 Department of Molecular Medicine and USF Health Byrd Alzheimer’s Research Institute, Morsani College of Medicine, University of South Florida, Tampa, FL USA; 7 Shanghai Institute of Cardiovascular Diseases, Department of Cardiology, Zhongshan Hospital Fudan University, Shanghai, China; 8 State Key Laboratory of Cardiovascular Diseases, Zhongshan Hospital, Fudan University, Shanghai, China

**Keywords:** Senescence, Diseases

## Abstract

Cellular senescence is a process in which the cell cycle becomes permanently arrested, thereby inhibiting cell division, proliferation and growth. Various cellular stresses, such as DNA damage, telomere shortening and oxidative stress, can trigger cellular senescence. Physiologically, cellular senescence contributes to tissue development, repair and critical biological processes such as embryogenesis, whereas, pathologically, it plays a key role in diverse disease subsets. To this end, elucidating the underlying mechanisms and molecular regulation of senescence is crucial. Here, in this Review, we explore recent key findings on cellular senescence in experimental and human disease models, focusing on its molecular mechanisms, regulation and future research directions to advance the field and facilitate therapeutic translation.

## Cellular senescence mechanisms impacting health and disease

Cellular senescence is a natural process in which cells stop dividing in response to stress, such as DNA damage. However, it can also contribute to diseases such as idiopathic pulmonary fibrosis (IPF), a lung condition with unknown causes. Researchers found that a protein called YTHDC1 can delay cellular senescence and reduce lung fibrosis by improving DNA repair in cells. They studied this in mice and found that increasing YTHDC1 levels helped to repair DNA and reduced fibrosis. The study used experimental methods to overexpress YTHDC1 in mouse lung cells, showing its potential to reverse senescence. The results suggest that targeting YTHDC1 could be a new way to treat IPF. However, more research is needed to confirm these findings and explore their safety in humans. In conclusion, understanding cellular senescence’s role in diseases such as IPF could lead to new treatments.

This summary was initially drafted using artificial intelligence, then revised and fact-checked by the author.

## Overview of cellular senescence

Cellular senescence is a biological process observed in eukaryotic and some prokaryotic cells^1,2^. It refers to the permanent arrest of the cell cycle in response to exogenous or endogenous stimuli, such as persistent or unresolvable DNA damage and telomere shortening. This process prevents the proliferation of faulty or damaged cells and inhibits their inheritance^3–5^. As a tumor suppressor mechanism, cellular senescence protects healthy cells from malignant transformation^6,7^. However, in already transformed malignant cells, cellular senescence may paradoxically promote tumor progression through diverse mechanisms^8,9^. Beyond cancer, cellular senescence plays a role in tissue development, remodeling, regeneration, wound healing and repair, and embryogenesis^4,10,11^. These effects are largely attributed to the senescence-associated secretory phenotype (SASP), characterized by the secretion of cytokines, chemokines, proteases, growth factors, immune modulators and matrix metalloproteinases^12–14^. For instance, SASP can promote vascularization and immunosuppression, by suppression of CD8^+^ T cells through TGF-β and IL-10 secretion, thereby facilitating tumor growth, metastasis, invasion and drug resistance^8,15,16^. Conversely, in tissue regeneration, SASP factors activate and recruit phagocytic and progenitor or stem cells^17^. Morphologically, senescent cells exhibit distinct features such as increased size and a flattened shape^18,19^.

Advanced aging leads to cumulative cellular damage, including oxidative stress and DNA damage, which, in turn, activates cellular senescence. This Review will first summarize the general mechanisms of cellular senescence, then discuss recent key findings on its molecular mechanisms and regulations and, finally, outline research directions in related human diseases.

## General mechanisms of cellular senescence

**Fig. 1:**
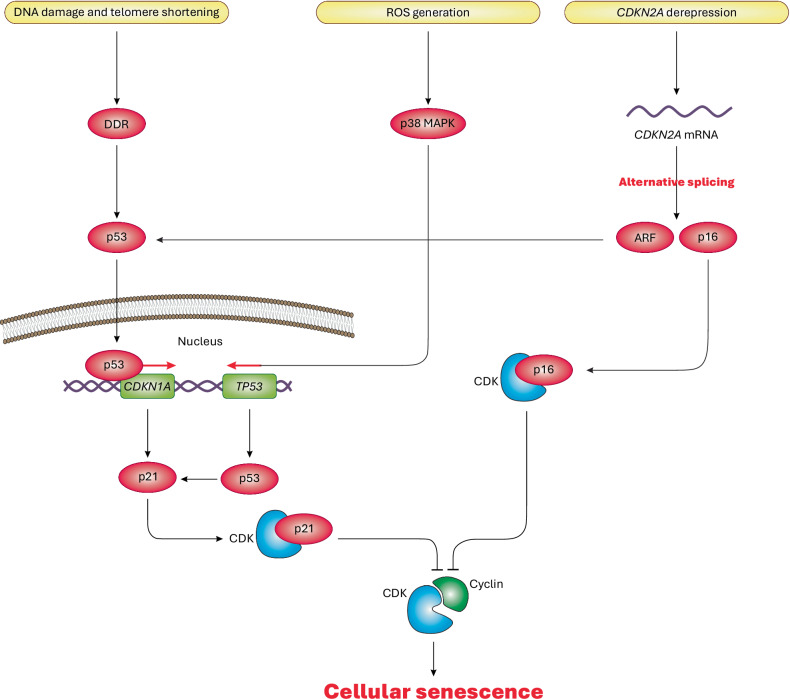
General mechanisms of cellular senescence. As discussed in the text, DNA damage and telomere shortening trigger the DNA damage response (DDR), thus inducing p53 transcription factor to translocate to the nucleus and transactivate *CDKN1A* gene, ultimately producing p21 (a CDK inhibitor). Subsequently, p21 binds to specific CDK proteins to prevent their binding with cyclin proteins, leading to cell cycle arrest and cellular senescence. Meanwhile, aging-associated *CDKN2A* derepression activates alternative splicing of *CDKN2A* mRNA, which produces two distinct proteins: ARF and p16. ARF activates p53 signaling, leading to the activation of p21, while p16 (another CDK inhibitor) can directly bind to specific CDK proteins to prohibit the formation of CDK–cyclin complexes, resulting in cellular senescence. Finally, increased ROS production can turn on p38 MAPK signaling, leading to transcriptional activation of *TP53*/p53 and heightened function of the p53–p21–CDK axis, ultimately ceasing cell cycle and inducing cellular senescence. Of note, in this figure, CDK refers to the CDK protein family. In detail, p21 can specifically bind to CDK2 and also CDK1, CDK4 and CDK6, while p16 can specifically bind to CDK4 and also CDK6. Although not displayed in the figure, retinoblastoma protein (Rb) also participates in cellular senescence by inhibiting the activity of early region 2 binding factor (E2F) transcription factors (involved in cell proliferation) and forming heterochromatin in the promoter region of their target genes^138^.

The stimuli that induce cellular senescence are diverse and increasingly recognized^20^. Most of these stimuli share similar mechanisms of action (Fig. 1), instigating signaling pathways that ultimately activate p53 (tumor protein P53) and various cyclin-dependent kinase (CDK) inhibitors, such as cyclin-dependent kinase 4 inhibitor B (p15/*CDKN2B*), cyclin-dependent kinase inhibitor 2A (p16/*CDKN2A*), cyclin-dependent kinase inhibitor 1A (p21/*CDKN1A*) and cyclin-dependent kinase inhibitor 1B (p27/*CDKN1B*). Such activation inhibits CDK and their complex formation with cyclins (CDK–cyclin), resulting in cell cycle arrest and cellular senescence^4^.

Telomeres are DNA sequences with associated proteins at chromosome ends, preventing tangling and degrading during DNA replication^21^. However, successive cell divisions often fail to maintain telomere length, leading to telomere shortening, a key driver of aging and cellular senescence^22,23^. Telomere shortening induces DNA damage, triggering the DNA damage response and activating DNA damage kinases, such as checkpoint kinase 2 (CHK2), checkpoint kinase 1 (CHK1), ataxia telangiectasia and Rad3 related (ATR), and ataxia telangiectasia mutated (ATM). These kinases phosphorylate and activate proteins involved in cell cycle regulation, including p53 (ref. ^24^). As a transcription factor, p53 upregulates *CDKN1A*, encoding p21, which inhibits CDKs (cyclin-dependent kinases 1, 2, 4 and 6) disrupting CDK–cyclin complexes and leading to G1- and S-phase cell cycle arrest^25,26^ (Fig. 1).

*CDKN2A* mRNA encodes two tumor suppressors, p16 and ARF (tumor suppressors), through alternative splicing^27^. Typically, in young tissues, *CDKN2A* expression is low or repressed, but aging induces its upregulation, a process known as *CDKN2A* locus derepression^28^. As a result, p16 and ARF tumor suppressors are upregulated and activated. In turn, p16 binds to and inhibits CDK4 and CDK6, whereas ARF stabilizes p53 by inhibiting mouse double minute 2 homolog (MDM2), an E3 ubiquitin-protein ligase responsible for p53 degradation (Fig. 1)^29,30^. Through these mechanisms, both p16 and the ARF–p53 axis disrupt CDK–cyclin complexes, effectively arresting the cell cycle.

Increased oxidative stress, linked to DNA damage, oncogene activation and external factors, is a key inducer of cellular senescence. This explains why antioxidant treatment can delay or inhibit cellular senescence^31–33^. Reactive oxygen species (ROS) activate p38 mitogen-activated protein kinase (MAPK) signaling, which upregulates *TP53*/p53 transcription, leading to increased *CDKN1A*/p21 expression. This blocks CDK–cyclin complexes, thereby halting the cell cycle^34^. Similarly, activation of various oncogenes (including over 50 members, such as RAS genes) has been shown to induce cellular senescence. This oncogene-induced cellular senescence probably serves as a mechanism to prevent tumorigenesis^35^. Several prominent reviews have discussed this topic in detail^35–37^. In addition, the loss of tumor suppressor genes such as phosphatase and tensin homolog (*PTEN*), neurofibromatosis type 1 (*NF1*) and von Hippel-Lindau (*VHL*) also induces cellular senescence as an antitumorigenic response^38,39^. Despite these established drivers, cellular senescence mechanisms remain highly complex and context dependent across different cell types, models and diseases. Below, we highlight recent key findings that enhance our understanding of this cellular process and its implications for disease and therapy.

## Cellular senescence in idiopathic pulmonary fibrosis and the role of YTHDC1

Idiopathic pulmonary fibrosis (IPF) is characterized by interstitial fibrosis in the lung with unknown causes^40–42^. Advanced aging has been shown to promote IPF^43–45^. For instance, aging is accompanied by cumulative DNA damage in pulmonary cells^46–48^. DNA damage is a profound trigger of cellular senescence^49–51^, exacerbating IPF^52–54^. Hence, pulmonary fibrosis and its subtypes, such as IPF, place cellular senescence at the core of their pathophysiology.

**Fig. 2:**
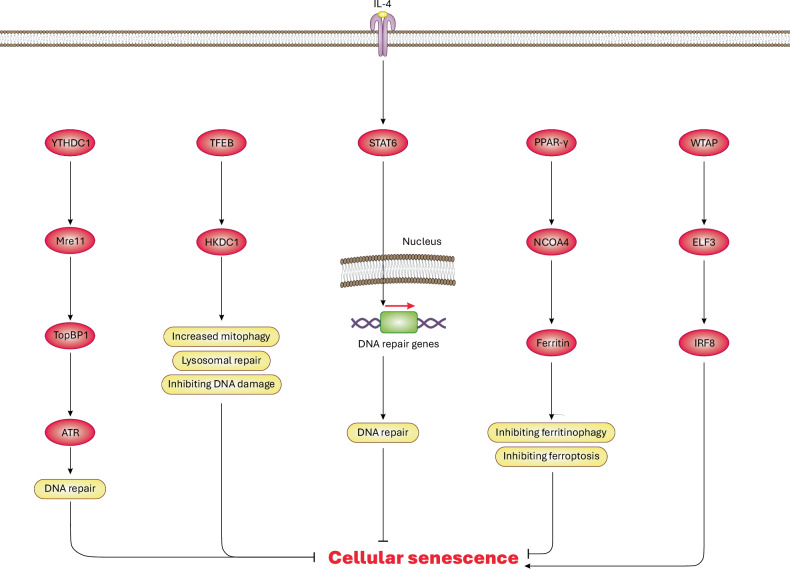
Regulation of cellular senescence in various types of mammalian cells. As comprehensively discussed in the text, YTHDC1 can induce DNA repair mechanisms through activation of the YTHDC1–Mre11–TopBP1–ATR axis, thereby inhibiting DNA damage and cellular senescence. Besides, TFEB transcription factor may combat cellular senescence by inducing activation and transcription of *HKDC1*/HKDC1, resulting in facilitated mitophagy, lysosomal repair and prevention of DNA damage. Moreover, IL-4-mediated activation of STAT6 signaling can also lead to transcription of DNA repair genes, thus reversing DNA damage and cellular senescence. Meanwhile, activation of the PPAR-γ–NCOA4 axis preserves ferritin in the cytosol, leading to inhibition of ferritinophagy and proferroptotic signaling, to lower iron overload and blockade of cellular senescence. Conversely, WTAP serves as a key subunit of the m^6^A methyltransferase complex to activate the ELF3–IRF8 axis, which instigates cellular senescence (in skin cells). Of note, the mechanisms illustrated here are not common in all cell types but rather specific to certain cell or animal models under different diseases or conditions as discussed in the main text.

YTH *N*^6^-methyladenosine RNA binding protein C1 (*YTHDC1*/YTHDC1) is an RNA-binding protein that binds to *N*^6^-methyladenosine (m^6^A) and is primarily expressed in pulmonary alveolar epithelial type 2 (ATII) cells^55,56^. Recently, it was shown that *YTHDC1* was downregulated in pulmonary fibrosis in ATII cells^52^. Experimental study of mouse ATII cells showed that exogenous overexpression of *Ythdc1* reversed cellular senescence and neutralized fibrosis independent of m^6^A binding, while its deficiency promoted IPF progression in mice^52^. Mechanistically, YTHDC1 mediates the interaction between Mre11 (MRE11 homolog, double-strand break repair nuclease) and DNA topoisomerase II binding protein 1 (TopBP1) proteins, leading to ATR activation and enhanced DNA damage repair. This delays cellular senescence and alleviates fibrosis^52^. Generally, Mre11 is essential for processing double-strand DNA breaks^57^, while TopBP1 acts as a scaffold protein, recruiting key DNA repair factors^58^. ATR localizes to DNA lesions and regulates DNA repair by phosphorylating downstream targets such as Chk1 (ref. ^59^). Thus, the YTHDC1–Mre11–TopBP1–ATR axis plays a crucial role in DNA repair. Several reviews have explored DNA repair mechanisms in more detail^60–62^. Collectively, these findings suggest that YTHDC1 can prevent or delay cellular senescence by enhancing DNA repair. Pulmonary-specific genetic upregulation of YTHDC1 could be a potential therapeutic strategy for IPF (Fig. 2), although further studies are needed to validate its safety and clinical applicability in IPF.

Although cellular senescence is fundamentally defined by cell cycle arrest, understanding its molecular links to pulmonary fibrosis and IPF pathology is crucial. However, Zhang and colleagues^52^ largely overlooked this aspect. Their findings suggest that cellular senescence may promote fibrosis through yet unexplored mechanism(s), underscoring the need for further studies to clarify the mechanistic connection between cellular senescence and fibrosis.

Nonetheless, evidence suggests that senescent cells primarily drive the fibrotic process by secreting cytokines, chemokines and growth factors, collectively known as the SASP^63^. Specifically, senescent ATII cells release SASP factors that trigger profibrotic responses in nearly alveolar macrophages and fibroblasts, promoting pulmonary fibrosis^64–66^. However, further studies are needed to fully elucidate the impact of cellular senescence on pulmonary fibrosis and IPF. Moreover, the role of YTHDC1 in cellular senescence remains largely unexplored in non-IPF conditions, highlighting the need for similar research in other non-IPF disease contexts.

Building on preclinical findings, pilot studies have explored the link between cellular senescence and IPF in humans. One study investigated the therapeutic potential of senolytics—drugs that selectively target and eliminate senescent cells—in patients with IPF^67^. In this open-label trial, 14 patients with stable IPF received intermittent dasatinib plus quercetin (DQ) (1,250 mg/day of Q and 100 mg/day of D for 3 weeks, 3 days per week), with assessments of senolytic retention, completion rates and SASP modulation^67^. The findings revealed that the physical function of patients was significantly improved in tasks including walking, chair stands and gait speed^67^. Mild-to-moderate adverse effects, such as skin bruising or irritation, gastrointestinal discomfort and respiratory symptoms were noticed, but no severe adverse events occurred^67^. While the effects of DQ on plasma SASP factors were inconclusive, correlations between proinflammatory cytokines, matrix modulating factor and microRNAs with functional outcomes were observed^67^. This open-label pilot study provided the first clinical evidence that application of DQ senolytics may improve physical function in patients with IPF with manageable side effects, warranting larger randomized controlled trials in IPF senotherapy^67^. In line with this, another randomized placebo-controlled trial (phase I, RCT: NCT02874989) assessed the safety of DQ senolytic therapy in 12 patients with IPF aged 50 years or above^68^. Participants were blinded and randomized (1:1) to receive either DQ senolytic (1,250 mg/day of Q and 100 mg/day of D for three consecutive days per week) or a placebo^68^. The treatment was well tolerated, with no serious adverse effects reported, although some patients experienced anxiety and sleep disturbances^68^. Overall, these findings suggest that intermittent DQ therapy is feasible and well tolerated in patients with IPF^68^. However, larger-scale prospective studies are needed to assess its efficacy and long-term safety in alleviating IPF symptoms.

## Lysosomal and mitochondrial dysfunction: TFEB–HKDC1 in cellular senescence

Although not fully understood, cellular senescence is closely associated with the simultaneous dysfunction of both lysosomes and mitochondria^69^. Senescent cells, for instance, exhibit elevated levels of ROS, which can trigger mitochondrial DNA damage, further exacerbating ROS production. This creates a vicious cycle, amplifying DNA damage and accelerating the progression of cellular senescence^70^. In addition to mitochondrial dysfunction, senescent cells display characteristic lysosomal abnormalities, including a dramatic increase in size and number, altered enzymatic content, membrane permeabilization and pH neutralization^71^. Conversely, lysosomal dysfunction is associated with impaired mitochondrial biogenesis and turnover, resulting in excess ROS production, which, in turn, damages lysosomes, continuing this harmful feedback loop^69^.

Abundant evidence suggests that the transcription factor EB (TFEB) plays a key role in regulating mitochondrial and lysosomal biogenesis and function^72,73^. A recent study demonstrated that hexokinase domain containing protein-1 (*HKDC1*/HKDC1) is a direct target of TFEB, as shown by transcriptome and the chromatin immunoprecipitation followed by quantitative polymerase chain reaction (ChIP–qPCR) analyses. HKDC1 is localized on the mitochondrial outer membrane^73^. Generally, HKDC1 regulates mitochondrial membrane potential and glucose uptake, thus linking glycolysis to mitochondrial ATP generation^74^. During organelle stress in both mitochondria and lysosomes, TFEB induces upregulation of *HKDC1*/HKDC1, which stabilizes PTEN-induced kinase 1 (PINK1) on the mitochondrial membrane, resulting in the induction of the PINK1–Parkin-mediated mitophagy. This process removes damaged mitochondria and enhances their turnover^73^. In addition, HKDC1 contributes to lysosomal repair by interacting with mitochondrial voltage-dependent anion channels, favoring contact formation between mitochondria and lysosomes^73^. Of note, all these effects occur independently of HKDC1’s role in glucose metabolism. However, in mice, *Hkdc1* deficiency or loss-of-function mutation exacerbated mitochondrial and lysosomal impairment, thereby accelerating DNA damage, leading to induction of cellular senescence. Therefore, the activation of the TFEB–HKDC1 axis indicates an inhibitory role in cellular senescence by orchestrating mitochondrial mitophagy and lysosomal repair (Fig. 2).

Mitophagy is a unique type of autophagy that removes and degrades long-lived or damaged mitochondria through autophagosomal engulfment and the lysosomal degradation pathway^75–79^. The PINK1–Parkin axis is a canonical pathway of mitophagy and, on the basis of these findings, it may act as a mechanism to suppress cellular senescence, making it a therapeutic target for diseases driven by cellular senescence. Future studies should further explore the interplay between cellular senescence and mitophagy, with a focus on elucidating the underlying molecular mechanisms in greater detail.

In addition to the abovementioned studies, key research on aging-associated neurobiology has shown that cytosolic and nuclear levels of TFEB were significantly decreased in the hippocampus and frontal cortex of aged mice^80^. To investigate its role, transgenic mice with ectopic *Tfeb* expression in cortical and hippocampal neurons were generated. This led to a marked increase in mitochondrial transcription factor A (mtTFA), peroxisome proliferator-activated receptor-gamma coactivator (PGC-1α) and mitochondrial abundance^80^. Besides, *Tfeb* expression was accompanied by decreased biomarkers of cellular senescence, thereby facilitating memory skills in mice^80^. These findings highlight the anti-aging and antisenescence roles of TFEB in hippocampal and cortical neurons, supporting its potential as a therapeutic target for preventing senescence-associated neurodegenerative conditions such as age-related cognitive decline. Further studies are needed to elucidate the molecular mechanisms underlying TFEB’s regulation of mtTFA and PGC-1α in neuronal senescence^80^.

Age-related hearing loss (ARHL) also involves TFEB-regulated cellular senescence^81^. Oxidative-stress-induced DNA damage and cellular senescence in the cochlea are perceived to exacerbate ARHL, while autophagy activation may prevent cellular senescence, serving as a protective mechanism in cochlea cells^81^. Sodium arsenite (NaAsO_2_), an oxidative stress inducer, has been used to model cellular senescence in auditory cells^81^. Upon NaAsO_2_ exposure, oxidative DNA damage disrupts TFEB nuclear transportation impairing autophagy, lysosomal dysfunction and mitochondrial damage, ultimately promoting cellular senescence and SASP activation in auditory cells, which worsens ARHL^81^. These findings reinforce the protective role of TFEB against ARHL through autophagy activation and mitochondrial homeostasis. Additional studies are warranted to further elucidate TFEB-regulated molecular mechanisms in auditory cells and explore its therapeutic potential for mitigating ARHL and other aging-associated hearing disorders.

## Type 2 cytokines and regulation of cellular senescence in macrophages

Type 2 immunity is mediated by type 2 cytokines (for example, interleukin (IL)-4, IL-9, IL-13 and IL-5) that either protect the host or exert pathogenic activity^82^. In a recent study, the absence of type 2 cytokine signaling was linked to macrophage senescence, seemingly due to increased DNA damage^83^. However, activation of the IL-4–STAT6 (where STAT6 is signal transducer and activator of transcription 6) signaling pathway delayed cellular senescence in macrophages by upregulating DNA repair genes, including those involved in homologous recombination-mediated DNA repair and Fanconi anemia disease (a DNA repair disorder)^83^ (Fig. 2). However, *STAT6* deficiency induced nuclear release of DNA into the cytosol (a process that occurs upon DNA damage), leading to activation of cellular senescence. Administration of IL-4 has been shown to mitigate cellular senescence in macrophages, thereby extending health span in aged mice. Collectively, these findings suggest that activation of the IL-4–STAT6 axis in macrophages delays cellular senescence by enhancing DNA repair mechanisms^83^. However, this study did not investigate the STAT6-initiated DNA repair mechanisms and downstream signaling pathways in macrophages, highlighting a significant knowledge gap that warrants future research. Furthermore, while the study acknowledged the antisenescence and therapeutic potential of type 2 cytokines in diseases driven by cellular senescence, it interchangeably used the terms ‘immunosenescence’ and ‘cellular senescence’, which appears to be an inaccurate interpretation^83^. Immunosenescence refers to aging of the immune system, conferring diverse modes of immune dysfunction^15^. However, immunosenescence and cellular senescence are not interchangeable terms, as each denotes distinct cellular process. Immunosenescence occurs only in immune cells as a consequence of aging, causing decreased proliferation, dysfunction or altered function of immune cells^15,84^. Meanwhile, cellular senescence is a permanent arrest of cell cycle to halt proliferation of damaged or faulty cells (such as those with DNA damage) and can occur in any cell type, including immune cells^15,84^. For more detail, our recent review has substantially discussed molecular mechanisms and conceptualization of ‘immunosenescence’ and its difference to cellular senescence and other nearby concepts^85^. Other prominent reviews include those by refs. ^15,86,87^.

More research is needed before drawing definitive conclusions about the role of type 2 cytokines in regulating cellular senescence in disease contexts. Furthermore, the findings on the role of type 2 cytokines (for example, IL-4) in STAT6 activation and macrophage senescence regulation appear to be unique in the current literature, emphasizing the need for further high-quality studies to explore similar mechanisms in other immune cell types.

## Vascular smooth muscle cells, cellular senescence: the role of PPAR-γ–NCOA4

Senescence of vascular smooth muscle cells (VSMCs) is strongly linked to arterial stiffness and vascular remodeling, both of which are key contributors to the prevalence and progression of cardiovascular diseases^88,89^. Ferroptosis, meanwhile, is a nonapoptotic form of cell death characterized by iron accumulation in the cytosol and subsequent lipid oxidation, contributing to the pathogenesis of various diseases, including cardiovascular diseases^90–92^.

In a recent mouse study, proferroptotic signaling was shown to induce cellular senescence in VSMCs, which is associated with NAD^+^ depletion and arterial remodeling^93^. Mechanistic evidence further suggests that NAD^+^ depletion leads to impaired DNA repair, thereby triggering cellular senescence^94^. However, genetic or pharmacological modulations of proferroptotic signaling—such as reducing iron overload and oxidative stress—was found to prevent cellular senescence in VSMCs, leading to reduced vascular stiffness and mitigation of aneurysm formation in mouse abdominal arteries^93^. Inhibition of proferroptotic signaling increased nuclear transportation of peroxisome proliferator-activated receptor-γ (PPAR-γ) into the cytosol, thereby suppressing the formation of the nuclear receptor coactivator 4 NCOA4)–ferritin complex^93^. The NCOA4–ferritin complex is well perceived to drive ferritinophagy, a specific type of autophagy that selectively engulfs ferritin after its binding to NCOA4, leading to lysosomal degradation and clearance of ferritin^95,96^. As a result, ferritin increases in the cytosol to alleviate iron overload, thus preventing lipid peroxidation, ferroptosis and cellular senescence^93^. Collectively, these findings denote that activation of the PPAR-γ–NCOA4–ferritin axis averts cellular senescence in vasculature by inhibiting ferritinophagy and proferroptosis signaling (Fig. 2).

However, this study fails to elucidate the underlying molecular mechanisms and cellular signaling pathways that link the proferroptotic state—characterized by increased ferritinophagy, iron overload and lipid peroxidation—to the induction of cellular senescence. Hence, additional studies are needed to unravel the intricate relationship between these processes and the cellular predisposition to senescence. Moreover, while cellular senescence of vascular cell types (for example, VSMCs) appears to have pathological consequence on arterial structure and vascular function, compelling evidence is still lacking to mechanistically explain how cellular senescence drives these pathologies. In general, SASP is widely recognized as a key contributor to cell senescence-driven pathology. However, relying solely on SASP as an explanatory mechanism may be an oversimplification, underscoring the need for more comprehensive mechanistic insights.

## Skin tissues, cellular senescence: m^6^A modification and WTAP–ELF3–IRF8

m^6^A modification, as briefly discussed above, is the most prevalent posttranscriptional RNA modification, regulating various cellular processes and human diseases^97–99^. Growing evidence suggests that m^6^A modification plays a crucial role in the aging process and aging-associated diseases, including skin aging^100^. A recent in vivo study utilizing proteomics analysis revealed that Wilms’ tumor 1-associating protein (*WTAP*/WTAP) expression is closely associated with cellular senescence in skin tissues and human dermal fibroblasts (HDFs)^101^. In line with this, both aged skin cells and HDFs exhibited upregulated *WTAP*/WTAP expression, whereas *WTAP*/WTAP knockdown reversed senescence^101^. Functionally, WTAP serves as an essential subunit of the m^6^A methyltransferase complex, facilitating the recruitment of the complex to its target mRNAs^102^. Specifically, this study found that WTAP directly targets and binds to E74 like ETS transcription factor 3 (*ELF3*) mRNA, causing its m^6^A modification and increased expression^101^. Subsequently, activated ELF3 transcription factor binds to the promoter region of interferon regulatory factor 8 (*IRF8*) gene, promoting its transcription and protein expression, to provoke cellular senescence and SASP, ultimately exacerbating skin aging in mice^101^.

Collectively, these findings highlight the key role of m^6^A modification and the WTAP–ELF3–IRF8 axis in driving skin and HDF senescence and aging (Fig. 2). Hence, therapeutic approaches may benefit from targeting this axis and manipulating m^6^A modification of relevant genes for anti-skin-aging strategies. However, current experimental evidence remains insufficient for clinical application. For instance, the mechanistic role of IRF8 in cellular senescence remains unclear, warranting further investigation. In addition, similar to other studies, Zhou and colleagues^101^ focused primarily on senescence induction but overlooked subsequent events contributing to skin aging. Although SASP is widely recognized as a primary driver of skin aging, further mechanistic studies are needed to substantiate this link.

While WTAP drives senescence in skin aging, evidence suggests that it plays an antisenescence role in other tissues. For instance, *WTAP* overexpression in the preosteoblast MC3T3-E1 cell line significantly promoted m^6^A modification of specificity protein 1 (*SP1*) mRNA, increasing its stability^103^. Consequently, SP1 bound to the bone morphogenetic protein 2 (*BMP2*) promoter, upregulating its expression, which promoted osteoblast differentiation and inhibited senescence^103^. These findings suggest that activation of the WTAP–SP1–BMP2 signaling pathway delays cellular senescence of osteoblasts and is implicated in senile osteoporosis. However, further studies are needed to elucidate BMP2’s antisenescence role in osteoblasts. Moreover, similar basic studies should explore the regulatory role of the WTAP in senescence across other tissues and/or disease subsets.

## Dual role of cellular senescence in cancer

Cellular senescence plays a dual role in cancer, acting as both a tumor-promoting and tumor-suppressive mechanism^7,104^. As previously discussed, cellular senescence in healthy cells prevents malignant transformation and tumorigenesis by halting the proliferation of precancerous cells (for example, those with unresolvable DNA damage or activated oncogenes). However, once cancer has developed, senescence activation within tumor tissues may paradoxically promote tumor growth and drug resistance through complex and poorly understood mechanisms^7,105,106^. In re-malignant cells, cellular senescence serves as a tumor-suppressive mechanism by permanently arresting the cell cycle, thereby inhibiting cell proliferation and exposing senescent cells to immune-mediated clearance^7,107,108^. However, in established tumors, the regulation of cellular senescence involves a complex interplay of signaling pathways and molecular mechanisms, many of which remain incompletely understood, highlighting the need for further research. While the antitumorigenic role of cellular senescence is well documented, the mechanisms underlying its protumorigenic effect remain largely unexplored. The most prominent hypothesis suggests that SASP creates an immunosuppressive microenvironment, skewing the immune system in favor of tumorigenesis and cancer progression^109^. Several key reviews have discussed the role and implication of cellular senescence in cancer in detail^8,9,108^. Recent studies have explored the molecular mechanisms of cellular senescence in cancer. For instance, in a senescence model of colorectal cancer cells, *N*^6^-adenosine-methyltransferase 70 kDa subunit (METTL3) was upregulated, leading to N^6^-methylation of *CDKN2B* mRNA, which increases its stability, expression and protein levels^110^. Consequently, CDKN2B inhibited CDK4/CDK6, inducing cell cycle arrest and cellular senescence, a process linked to colorectal cancer progression^111–113,110^. Furthermore, this study suggested that senescent cancer cells promoted M2 macrophage polarization, contributing to colorectal cancer progression^110^. Nonetheless, the precise mechanisms underlying METTL3-driven senescence in cancer remains largely obscure, warranting additional studies.

## Intrinsic disorder as a unifying feature among proteins of cellular senescence

**Fig. 3:**
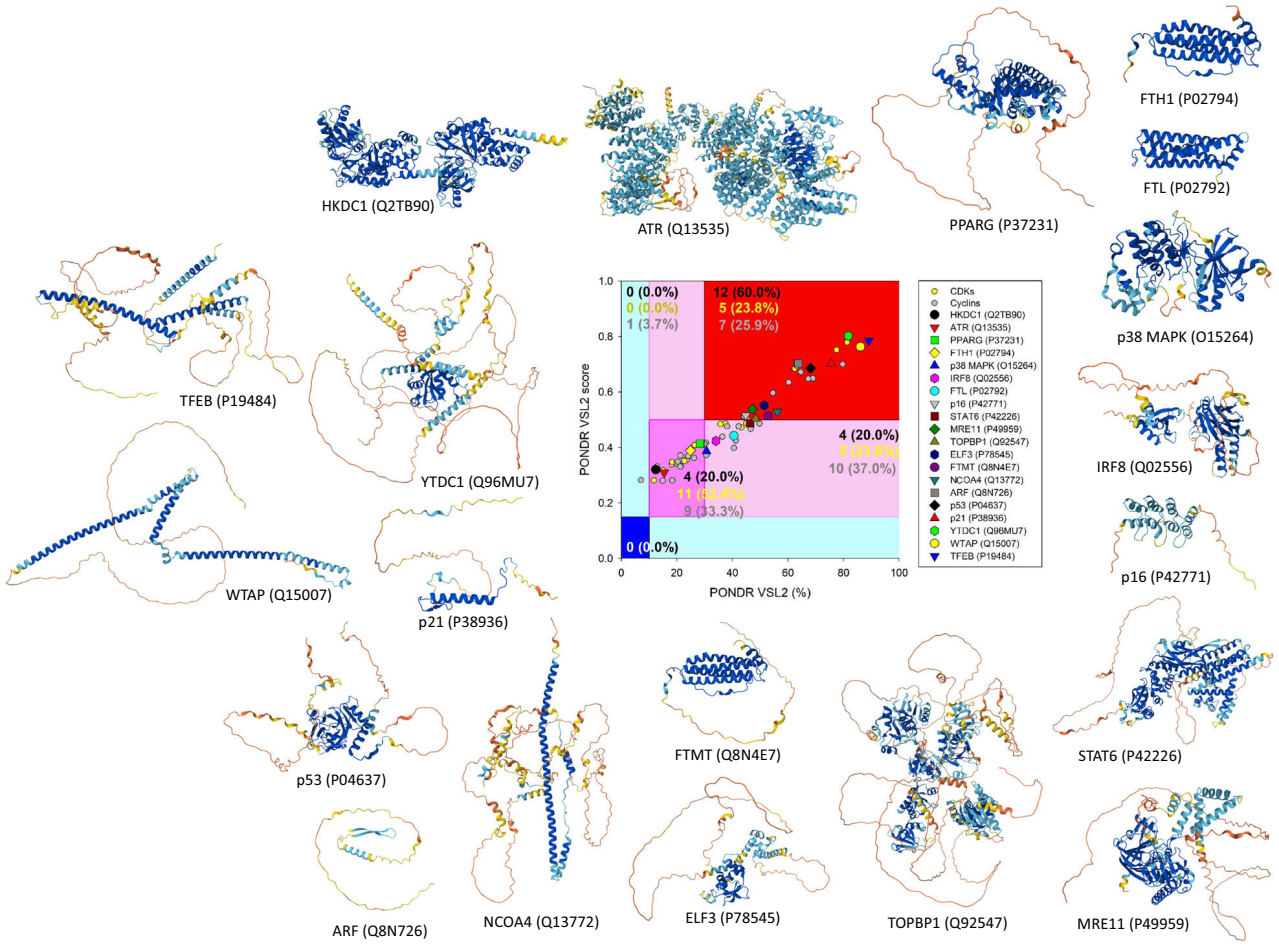
Intrinsic disorder status of human proteins controlling cellular senescence. The plot in the middle represents the results of the PONDR VSL2 score versus VSL2 PONDR^®^ (%) analysis. PONDR VSL2 (%) is the percent of predicted disordered residues (PPDR), that is, residues with disorder scores above 0.5. The PONDR VSL2 score is the average disorder score (ADS) for a protein. Color blocks indicate regions in which proteins are mostly ordered (blue and light blue), moderately disordered (pink and light pink) or mostly disordered (red). If the two parameters agree, the corresponding part of the background is dark (blue or pink), whereas light blue and light pink reflect areas in which the predictors disagree with each other. The boundaries of the colored regions represent arbitrary and accepted cutoffs for the ADS (*y* axis) and the PPDR *(x* axis). Here, residues, regions and proteins with an ADS of at least 0.5 are considered disordered, residues, regions and proteins with an ADS between 0.15 and 0.5 are considered ordered but flexible, and residues, regions and proteins with an ADS below 0.15 are considered ordered. Based on their PPIDR values, proteins are classified as ordered (PPIDR <10%), moderately disordered (10% ≤ PPIDR < 30%) and highly disordered (PPIDR ≥30%). The levels of intrinsic disorder in the sets of 21 human CDKs (UniProt IDs: Q9UQ88, Q9NYV4, Q9BWU1, Q96Q40, Q8IZL9, Q15131, Q14004, Q07002, Q00537, Q00536, Q00535, Q00534, Q00526, P50750, P50613, P49336, P24941, P21127, P11802, P06493 and O94921) and 27 human cyclins (UniProt IDs: Q8ND76, O60583, O60563, Q8N1B3, Q9H8S5, P22674, Q96S94, Q9UK58, O75909, Q5T5M9, Q6ZMN8, Q14094, P51946, Q16589, P51959, P41002, O96020, P24864, P30281, P30279, P24385, P24863, Q8WWL7, O95067, P14635, P20248 and P78396) are also presented for comparison as small yellow and gray circles, respectively. Data for this lot were retrieved by analyzing the corresponding protein sequences using the RIDAO platform^139^. The three-dimensional structures of these proteins were modeled by AlphaFold^116^. Structures are colored according to the per-residue model confidence score, *p*_LLDT_. Here, blue color corresponds to regions with *p*_LDDT_ > 90, which are modeled with high accuracy, cyan color shows well-modeled regions with *p*_LDDT_ between 70 and 90, yellow color corresponds to low-confidence regions with *p*_LDDT_ between 50 and 70, and orange color indicates regions with very low-confidence *p*_LDDT_ < 50, which often have a ribbon-like appearance and are probably intrinsically disordered.

Evidence presented here emphasizes that cellular senescence is regulated by proteins belonging to different functional classes. However, a key unifying feature among these functionally diverse proteins is their intrinsic disorder status. In agreement with a recent comprehensive analysis highlighting a significant presence of intrinsic disorder in aging-related proteins^114^, Fig. 3 shows that human proteins controlling cellular senescence are systematically enriched in intrinsic disorder. Specially, based on their intrinsic disorder content (Fig. 3, middle), all 20 proteins discussed in this Review are classified as disordered, with HKDC1, ATR, PPAR-γ and ferritin heavy chain being moderately disordered (their percent of predicted intrinsically disordered residues (PPIDR) ranges from 12.4% to 28.4%) and remaining proteins being disordered (that is, possessing PPIDR values of at least 30%). Furthermore, 12 of these proteins (p16, STAT6, MRE11, TOPBP1, ELF3, mitochondrial ferritin, NCOA4, ARF, p53, p21, YTDC1, WTAP and TFEB) are classified as highly disordered, characterized by mean disorder scores exceeding 0.5. In other words, 20% of the proteins in this set are moderately disordered, 20% are disordered and 60% are highly disordered, as indicated by their placement in the pink, light-pink and red areas of the PONDR VSL2 score versus PONDR^®^ VSL2% plot (Fig. 3). In addition, relatively high levels of disorder are observed in human CDKs and cyclins (Fig. 3, small yellow and gray circles). By comparison, in the human proteome, 0.41%, 5.07%, 33.67%, 21.01% and 39.84% proteins are highly ordered, ordered, moderately disordered, disordered and highly disordered, being positioned within the blue, cyan, pink, light-pink and red areas of a similar plot^115^. Further evidence of the abundant presence of intrinsic disorder in human proteins controlling cellular senescence is given by their three-dimensional structures modeled by AlphaFold^116^. In fact, most structural models shown in Fig. 3 contain regions with low and very low per-residue confidence scores, which is a reflection of their disordered nature.

The high intrinsic disorder content in these proteins is unsurprising, as intrinsic disorder is a defining feature of protein multifunctionality and binding promiscuity^117^. This concept aligns with the protein structure–function continuum model, which posits that proteins exist as dynamic conformational ensembles, encompassing multiple proteoforms with a broad spectrum of structural features and diverse functional potentials^118–121^. Furthermore, intrinsic disorder is crucial for many enzymatically catalyzed posttranslational modifications^122–124^, is tightly linked to alternative splicing^125^ and plays crucial roles in liquid–liquid phase separation defining biogenesis of multiple membrane-less organelles and biomolecular condensates^126–130^. Detailed consideration of the roles of intrinsic disorder in functioning of these proteins is outside the scopes of this Review. However, the interested reader can find the related discussions reported for several of these proteins (for example, PPAR-γ^131^, p16^132^, MRE11^133^, TopBP1^134^, NCOA4^135^, ARF^132^, p53^136^ and p21^137^).

## Conclusions

In summary, recent literature highlights the prominent findings regarding the underlying mechanisms and regulation of cellular senescence across various experimental models and diseases. Cellular senescence serves as a stopwatch for the cell cycle under severe stress conditions, such as DNA damage, oxidative stress and telomere shortening. Activation of the p53 transcription factor and CDK inhibitors, such as p21, alongside subsequent inhibition of the CDK–cyclin complexes, represents the primary mechanism of cellular senescence in mammalian cells. While the physiological and pathological impacts of senescent cells are largely mediated by unknown mechanisms, the SASP characteristic of senescent cells is expected to be a key driving force of these effects. Nonetheless, research in this area is still ongoing, with new discoveries emerging each year. However, the translational relevance of current findings on cellular senescence remains limited, and further studies are required to address remaining questions and fill existing gaps. In the studies mentioned above, we aimed to provide insights into new research directions and potential bottlenecks for therapeutic intervention targeting cellular senescence in related conditions. Given the complexity of cellular senescence and the accumulating evidence, a single review may not be sufficient to comprehensively address all aspects of this process. Therefore, additional experimental studies are necessary to illuminate the intricate mechanisms and processes involved in cellular senescence.

## Availability of supporting data

Not Applicable
